# Efficacy of a low-FODMAP diet in adult irritable bowel syndrome: a systematic review and meta-analysis

**DOI:** 10.1007/s00394-020-02473-0

**Published:** 2021-02-14

**Authors:** Anne-Sophie van Lanen, Angelika de Bree, Arno Greyling

**Affiliations:** 1 Division of Human Nutrition and Health, Wageningen University and Research, Wageningen, The Netherlands; 2 Unilever, Unilever Foods Innovation Centre, Bronland 14, 6708 WH Wageningen, The Netherlands

**Keywords:** Low-FODMAP diet, Irritable bowel syndrome, Exclusion diet, Gastrointestinal symptoms

## Abstract

**Purpose:**

This review provides an updated overview of observational and intervention studies investigating the effect of a low-FODMAP (fermentable oligo-, di- and monosaccharides, and polyols) diet (LFD) on gastrointestinal (GI) symptoms, quality of life (QoL), nutritional adequacy, and gut microbiome in irritable bowel syndrome (IBS) patients.

**Methods:**

We systematically searched available literature until October 2020 for studies that investigated the effect of LFDs on GI symptoms, QoL, nutritional adequacy, and the gut microbiome in IBS patients. The data were represented as standardized mean differences (SMD) for IBS severity, and as mean differences (MD) for IBS-QoL. Meta-analyses were performed for the quantitative analyses using random effects models with inverse variance weighing.

**Results:**

Twelve papers (nine parallel trials, three crossover studies) were included for the meta-analysis. The LFD reduced IBS severity by a moderate-to-large extent as compared to a control diet (SMD − 0.66, 95% CI − 0.88, − 0.44, *I*^2^ = 54%). When analyzing only studies that used the validated IBS-SSS questionnaire, a mean reduction of 45 points (95% CI − 77, − 14; *I*^2^ = 89%) was observed. Subgroup analyses on adherence, age, intervention duration, IBS subtype, outcome measure, and risk of bias revealed no significantly different results. The LFD also increased IBS-QoL scores, when compared with a control diet (MD 4.93; 95% CI 1.77, 8.08; *I*^2^ = 42%).

**Conclusions:**

The low-FODMAP diet reduces GI symptoms and improves quality of life in IBS subjects as compared to control diets. Future work is required to obtain definitive answers regarding potential long-term effects of such diets on nutritional adequacy and the gut microbiome.

**PROSPERO registration number:**

CRD42020175157.

**Supplementary Information:**

The online version contains supplementary material available at 10.1007/s00394-020-02473-0.

## Introduction

Irritable bowel syndrome (IBS) is a functional gastrointestinal (GI) disorder that is characterized by abdominal pain, bloating, and altered bowel habits [1]. It is the most commonly diagnosed GI disorder, estimated to affect approximately 11% of the global population [2], with an increased prevalence in women as compared to men [3]. IBS has repeatedly been demonstrated to both reduce quality of life (QoL) [4–6] and increase health care utilization [7–9], leading to a significant economic burden [8–10].

The complex pathophysiology of IBS is not yet fully understood, but is suggested to involve visceral hypersensitivity, low-grade digestive tract inflammation, changes in GI motility, gut microbiota, and the gut–brain axis [1, 11–15]. As a result of this, IBS treatments currently rely on multifactorial approaches that are primarily focused on treating symptoms [13, 14, 16, 17]. Both IBS patients and gastroenterologists have reported a strong association between consumption of specific foods and IBS-related symptoms [4, 18, 19], indicating the need for an effective dietary treatment strategy. As each IBS subtype presents itself with different symptoms, treatment should be based on IBS subtype and symptom severity [1]. The goal of treatment for IBS with predominantly diarrhea (IBS-D) is to reduce the excessive bowel movements, while treatment for IBS with predominantly constipation (IBS-C) will aim for regular bowel movements, each requiring different nutritional approaches [1]. Besides, general advice to IBS patients comprises eating healthily and in small portions, limiting intakes of gas-producing and fermentable foods, alcohol, fat, and spicy foods [1, 20]. Many patients also try diets like the gluten-free and lactose-free diet to relieve symptoms [19]. Yet, there is little evidence for the efficacy of these elimination diets in the absence of specific conditions like lactose or gluten intolerance or celiac disease, and therefore these diets are not generally recommended [19, 21, 22].

However, there is a growing body of evidence for the effectiveness of the low fermentable oligo-, di- and monosaccharides, and polyols (FODMAP) diet (LFD) in managing IBS symptoms [22, 23]. Currently, advisory bodies like the American College of Gastroenterology and the British Dietetic Association advise the LFD to be respectively first- and second-line treatment for IBS [24, 25]. The underlying hypothesis suggests that reducing the intake of these small, indigestible and often fermentable carbohydrates, reduces intestinal osmolarity and gas production; hence, helping to reduce GI symptoms [26, 27]. The LFD starts with a general phase that aims to eliminate all FODMAPs. If symptoms are successfully reduced within 6–8 weeks, specific groups of FODMAPs are reintroduced into the diet. This serves to identify which FODMAPs cause symptoms, so that patients can adapt a personalized long-term diet that effectively reduces IBS symptoms. Owing to its restrictive nature, however, there are concerns about the effect of the LFD on nutritional adequacy, intestinal microbiota, and health-related quality of life [28–31]. Therefore, the LFD should only be followed in consultation with a specialized dietitian.

Since the two most recent meta-analyses that were performed on the effect of an LFD on GI symptoms in IBS patients [22, 23], four new RCTs and two new cross-sectional studies have been published. The purpose of the current work is to provide an updated systematic review and meta-analysis of both observational and intervention studies that investigates the effect of a low-FODMAP diet, as compared to a control diet, on GI symptoms and quality of life in IBS patients.

## Methods

The protocol for this systematic review and meta-analysis was registered in the international prospective register of systematic reviews (PROSPERO, registration number: CRD42020175157), and conducted and reported in accordance with the Preferred Reporting Items for Systematic Reviews and Meta-Analyses (PRISMA) statement guidelines [32].

### Search strategy

We systematically searched the electronic databases PubMed/Medline, SCOPUS, and Web of Science until October 1st 2020 for English language records. Titles, abstracts, and keywords were searched for variations and combinations of the following terms: FODMAP(s), saccharides, oligosaccharide, disaccharide, monosaccharide, galacto-oligosaccharides, fructan(s), fructose, galactans, lactose, polyol(s), sorbitol, mannitol, xylitol, maltitol, sweetener(s), sweetening agent, IBS, irritable bowel syndrome, and irritable colon. Separate searches including additional terms related to gut microbiome and nutritional adequacy were also performed (full PubMed search syntaxes in the Supplementary Materials). Intervention and observational studies were included when they respectively examined the effect of the LFD or assessed the association between FODMAP content in the diet and GI complaints or IBS prevalence in adult human subjects with IBS diagnosed according to the Rome III or IV criteria [11, 33].

Papers were excluded when they had an unsuitable intervention (e.g., a co-intervention from which the effects of an LFD could not be distinguished) or control diet, were conducted in children, non-IBS patients or IBS patients with significant clinical co-morbidities, were conference abstracts, or when English text was unavailable. In the case of multiple papers referencing the same study, relevant data were extracted from both papers and included as a single study in the analysis.

### Screening and selection of trials

The systematic search was followed by a two-step screening and selection process. During the first step, titles, abstracts, and keywords of publications were screened separately by two of the authors (ASL and AG) to identify potentially eligible studies. During the second step, the full texts of these publications were examined to gauge eligibility based on the stated inclusion criteria. In cases of inter-reviewer disagreement, questions on study eligibility were resolved through consensus and consultation with the other co-author (AB).

### Outcome assessment

The primary outcome of interest was IBS symptom severity, preferably assessed by the widely used and validated IBS Severity Scoring System (IBS-SSS) [34]. The IBS-SSS questionnaire assesses the intensity of GI symptoms during a 10-day period and focuses on abdominal pain, distension, stool frequency and consistency, and interference with daily life. Each of these items is scored on a 0–100 visual analog scale, adding up to a total sum score of 0–500, with higher scores indicating more severe symptoms [34]. Studies using other measures of symptom severity, both validated measures and nonvalidated VAS and Likert scales, were included as well. When no assessment of the overall symptom severity was reported, abdominal pain was used as an outcome of interest [22].

The secondary outcomes of interest were quality of life, gut microbiome effects and impact on measures of nutritional adequacy. Quality of life was measured by the validated IBS-QoL questionnaire [35]. The IBS-QoL questionnaire consists of 34 questions regarding dysphoria, interference, body image, health worry, food avoidance, social reaction, sexual, relationships. The results are averaged and transformed to a 0–100 scale, with increasing scores indicating a better QoL [35]. Owing to heterogeneity in methodology and reporting of data, it was deemed inappropriate to conduct meta-analyses of the gut microbiome and nutritional adequacy data. These outcomes were therefore included as part of the qualitative analysis.

### Data extraction and quantification

Data extraction was performed by two authors (ASL, AG) and consisted of information on the year of publication, country of origin, study design, duration, intervention diet, control diet, adherence to the diets, number of cases, number of controls, total sample size, IBS diagnostic criteria, mean age and gender, and IBS subtype distribution. The means (mean value at the end of the intervention and end of control period, respectively) and standard deviations between symptom severity measures and IBS-QoL before and after intervention were collected. If no means and standard deviations were reported in the text, the data were extracted from tables or graphs (using a web-based plot digitizing tool [36]). When these data were not available and whenever possible, the 95% CIs and *P* values were used to calculate means and standard deviations [37]. Where median values and ranges were reported, they were converted to mean values and standard deviations according to the conversion formulas of Wan et al. and Luo et al. [38, 39]. This was done in one case [40]. Where no end values were reported, change from baseline data were used instead [41, 42]. Where insufficient data were available to calculate or extract the mean and standard deviation, the study was excluded from analysis [43].

### Data synthesis and statistical analysis

For the primary outcome, standardized mean differences (SMD) were calculated to allow comparison between the variety of outcome measures used in the studies, and to prevent unnecessary exclusion of study data. The SMD is a unitless measure that can be interpreted as a small, moderate or large magnitude of effect [44]. Meta-analyses were conducted using a random effects model with inverse variance weighing [45]. Where enough data were available (minimum of four studies per subgroup), the potential effects of predefined covariates (IBS subtype, intervention duration, sex, age) on the change in IBS severity measures were examined by means of subgroup analyses. The *I*^2^ statistic was inspected to assess the extent of possible heterogeneity with *I*^2^ values of 25, 50, and 75% considered to be low-, moderate-, and high-level heterogeneity respectively [46]. Data analysis was performed using Review Manager 5 (Version 5.4, Cochrane).

### Risk of bias assessment

Publication bias was investigated through visual inspection of funnel plots and Egger’s regression test (with *P* < 0.1 indicating asymmetry) [47]. The risk of bias in the included studies was assessed using the Cochrane Collaboration’s tool for assessing risk of bias [48]. For this purpose, seven different domains were considered: random sequence generation, allocation concealment, blinding of participants and personnel, blinding of outcome assessment, incomplete outcome data, selective reporting, and other sources of bias. For cross-sectional studies, we used an adapted version of the Newcastle–Ottawa quality assessment scale [49]. The assessments were carried out independently by two authors (ASL and AG), and differences resolved by consensus.

## Results

### Quantitative analysis

#### Included trial characteristics

**Fig. 1:**
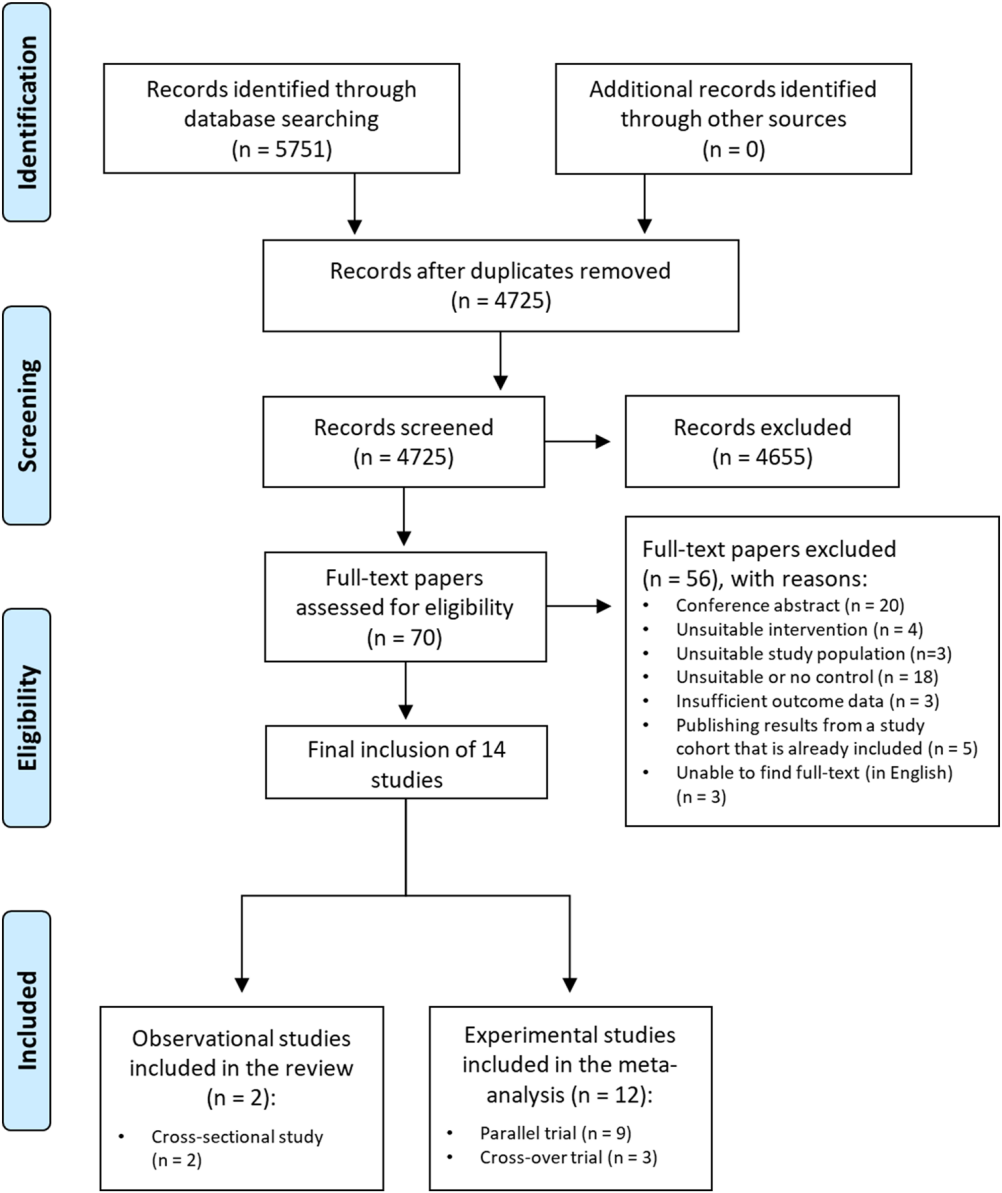
Preferred Reporting Items for Systematic Reviews and Meta-Analyses (PRISMA) flow diagram of the study selection procedure

A total of 5751 records was identified through database searching. After duplicate removal, 4725 records were screened, leading a full-text assessment of 70 studies. After exclusion of 56 studies, 14 original studies were included in the review (Fig. 1). Of these, 12 original parallel or crossover trials reported on IBS symptom severity outcomes (Table 1) and were included in the meta-analysis. The remaining two cross-sectional studies are described in Table 2. One post hoc analysis reported quality of life data from the same study population as a study that was already included. Relevant data were extracted, and the paper was excluded [50].

**Table 1:** Characteristics of experimental studies included in the meta-analysis

First author, year (country)	Study duration	Total case/controls^a^	Type of treatment: intervention vs. control	Age (years)^b^	Female (%)^b^	Predominant IBS subtype (%)^b^	Results
Bohn, 2015 [63] (Sweden)	4 weeks	33/34	Dietary education: LFD vs. traditional IBS diet (NICE and BDA)	42.5	81.3	IBS-M (47)	No significant difference in IBS-SSS was observed between the LFD and control group (246 vs. 23, *P* = 0.62)
Eswaran, 2016 [70] (USA)	4 weeks	43/39	Dietary education: LFD vs. traditional IBS diet (NICE)	42.6	70.7	IBS-D (100)	The mean abdominal pain score decreased to 3.4 in the LFD group vs. 4.4 in the mNICE group (*P* = 0.005), and the IBS- QoL score increased to 69.3 for the LFD group vs. 59.4 for the mNICE group (*P* value not reported)
Halmos, 2014 [61] (Australia)	42 days	30/30	Provided diets: LFD vs. typical Australian diet (4.4 g oligosaccharides and 2.6 g polyols/day)	41.0	71.1	IBS-C (43)	Subjects reported lower mean VAS-scores (0–100) for GI symptoms when on an LFD compared to control: 22.8 vs. 44.9 (*P* < 0.001)
Harvie, 2017 [52] (New Zealand)	3 months	23/27	Dietary education on LFD vs. no dietary education	41.8	86	IBS-D (64)	Subjects on the LFD had a lower mean IBS-SSS (128 vs. 206) and higher mean IBS-QoL (81 vs. 73) compared to control, after 3 months (*P* < 0.05 in both for improvement)
McIntosh, 2017 [64] (Canada)	21 days	18/19	Dietary education: LFD vs. HFD	50.9	86.5	IBS-M (62)	Mean IBS-SSS decreased to 208 in the LFD group vs. 290 in the control group (*P* = 0.01)
Ong, 2010 [40] (Australia)	4 days	15/15	Provided diets: LFD (9 g FODMAPs/day) vs. HFD (50 g/day)	40.8	73.3	IBS-C (47)	IBS symptom severity assessed by a self-rating Likert scale was reported to be lower during the LFD (median 2; range 0–7) than during HFD (6; 2–9)
Paduano, 2019 [53] (Italy)	12 weeks	34/28	Dietary education: LFD vs. balanced Mediterranean diet	28.6	83.3	IBS-D (52)	No significant differences were found between the LFD and control diet when looking at mean IBS-SSS (16 vs. 17, *P* = 0.44) and IBS-QoL (83 vs. 81, *P* = 0.27)
Patcharatrakul, 2019 [62] (Thailand)	4 weeks	30/32	Dietary education: personalized LFD vs. commonly recommended diet to reduce IBS symptoms	51.0	75.8	IBS-C (53)	The mean global IBS symptom severity score (VAS 0–100) after intervention was lower in the LFD group than the control group (38.5 ± 20 vs. 53.5 ± 19, *P* < 0.01)
Pedersen, 2014 [41] (Denmark)	6 weeks	42/40	Dietary education: LFD including personalized reintroduction vs. unchanged Danish/Western diet	34.6	76.8	IBS-D (45)	There was a significantly greater reduction in mean IBS-SSS in the LFD group than in the control group (133 vs. 34, *P* < 0.01). Mean IBS-QoL was not altered significantly (LFD: 8 vs. control: 0.1, *P* = 0.13)
Staudacher, 2012 [66] (UK)	4 weeks	16/19	Dietary education: LFD vs. habitual diet	35.1	35.1	NR	The mean overall symptom severity score (0–3 scale) after intervention was lower in the LFD group than in the control group (1.1 vs. 1.7, *P* < 0.002)
Staudacher, 2017 [69] (UK)	4 weeks	51/53	Dietary education: LFD vs. sham exclusion diet (comparable in number of restricted foods and difficulty)	34.4	68.6	IBS-D (67)	Mean IBS-SSS was significantly lower for patients on the LFD than the sham diet (173 vs. 224, *P* = 0.001). No significant difference was observed between the groups for IBS-QoL (72.4 vs. 70.6, *P* = 0.057)
Zahedi, 2018 [42] (Iran)	6 weeks	50/51	Dietary education: LFD (< 0.5 g of FODMAPs per meal) vs. traditional IBS diet (BDA)	37.5	50.5	IBS-D (100)	Mean IBS-SSS decreased to a greater extent in the LFD group compared to control (108 vs. 149.8, *P* = 0.002). No significant difference was observed between the groups for IBS-QOL (− 7.3 vs. − 5.35, *P* = 0.332)

The data are represented as mean value unless indicated otherwise

*BDA* British Dietetic Association; *FODMAP* fermentable oligo-, di-, monosaccharides and polyols; *HFD* high-FODMAP diet; *IBS-C* irritable bowel syndrome with constipation; *IBS-D* irritable bowel syndrome with diarrhea; *IBS-M* irritable bowel syndrome with mixed stool pattern; *IBS-QoL* irritable bowel syndrome-associated quality of life; *IBS-SSS* irritable bowel syndrome severity scoring system; *LFD* low-FODMAP diet; *NICE* National Institute for Health and Care Excellence; *NR* not reported

^a^Numbers are retrieved from per-protocol data

^b^Numbers are retrieved from intention-to-treat data

**Table 2:** Characteristics of observational studies included in the qualitative synthesis

First author, year (country)	Study design	Number of subjects	Diagnostic criteria	Age (years)	Female (%)	Predominant IBS subtype (%)	Quality assessment^a^ (number of stars^b^)	Results
Lee, 2019 [18] (South Korea)	Cross-sectional	393	Validated modified Korean Rome III	49.4	61.8	IBS-D (43.6)	Poor (3)	High-FODMAP foods were reported by 43.5% of controls^c^ and 63.4% of IBS subjects to induce GI symptoms
Pourmand, 2018 [51] (Iran)	Cross-sectional	3362 (number of confirmed IBS cases NR)	Unvalidated modified Persian Rome III	NR	NR	NR	Good (7)	No significant association was found between adherence to the LFD and IBS prevalence

The data are represented as mean value unless indicated otherwise

*FODMAP* fermentable oligo-, di-, monosaccharides, and polyols; *IBS-D* irritable bowel syndrome with diarrhea; *LFD* low-FODMAP diet; *NR* not reported

^a^According to an adapted Newcastle–Ottawa scale for cross-sectional studies [48]

^b^On a scale from 0 to 10

^c^The control group comprised of symptomatic and nonsymptomatic subjects

A total of 772 subjects took part in the nine parallel and three crossover trials that investigated the effect of an LFD on GI symptoms in IBS patients. The number of participants per study ranged from 30 to 104. The study duration ranged from 4 days to 3 months. The mean age ranged from 29 to 51 years. Two studies were controlled diet interventions that provided almost all food to subjects during the intervention. Subjects in the remaining ten studies received dietary education as an intervention. The control diets, provided or prescribed, comprised a traditional IBS diet (*n* = 4), the subjects habitual diet (*n* = 2), typical diet for the country where the study was carried out (*n* = 2), high-FODMAP diet (*n* = 2), balanced Mediterranean diet (*n* = 1), or a sham exclusion diet specifically designed for the study (*n* = 1).

#### Effect of LFD on GI symptoms in IBS patients

**Fig. 2:**
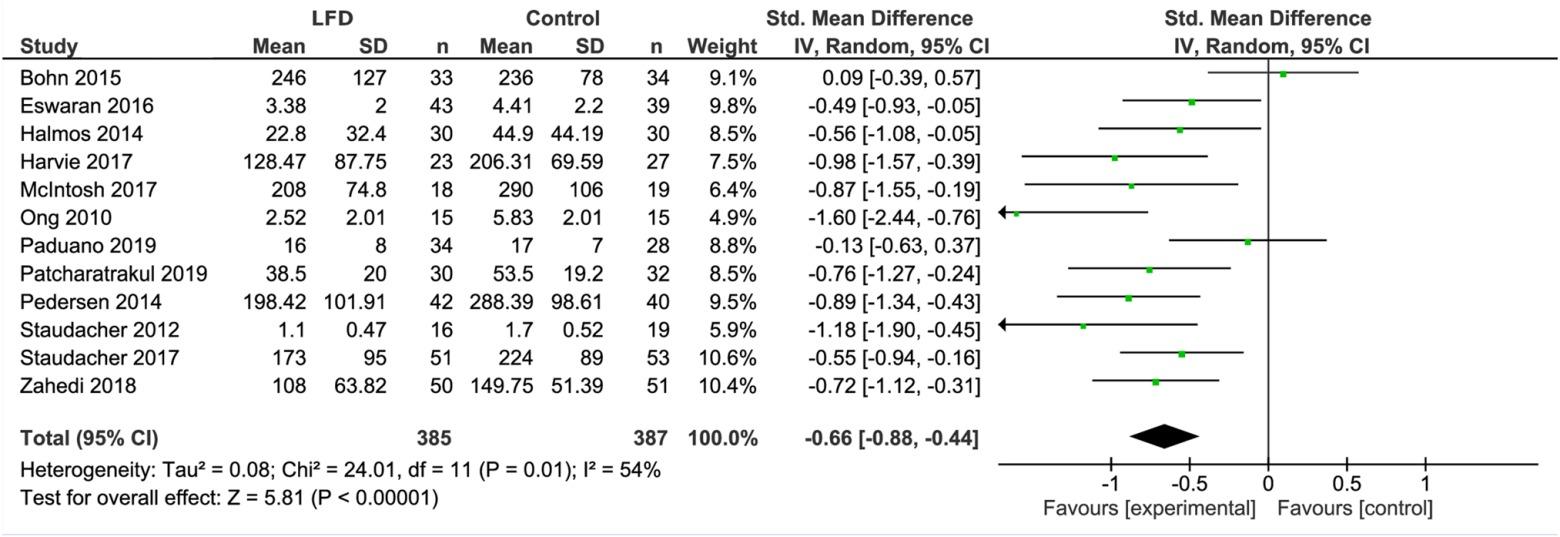
Forest plot showing standardized mean differences for IBS severity outcome measures

**Fig. 3:**
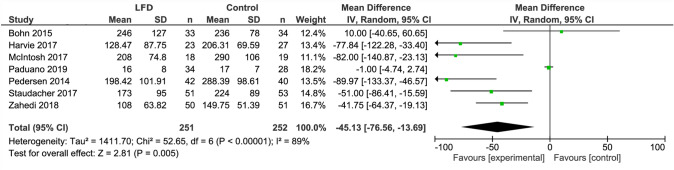
Forest plot showing mean IBS-SSS scores for studies that used IBS-SSS as outcome

The LFD was found to reduce IBS severity by a moderate to large extent as compared to a control diet (SMD − 0.66, 95% CI − 0.88, − 0.44, *I*^2^ = 54%) (Fig. 2). When analyzing studies that used IBS-SSS only, a mean reduction of 45 points (95% CI − 76.56, − 13.69; *I*^2^ = 89%) was observed (Fig. 3).

One of the observational studies included in the qualitative analysis observed a larger proportion of IBS subjects to report high-FODMAP foods to induce GI symptoms, as compared to control subjects (63.4% vs. 43.5% respectively) [18] (Table 2). The other observational study reported no association between adherence to the LFD and IBS prevalence [51] (Table 2).

#### Effect of LFD on QoL in IBS patients

**Fig. 4:**
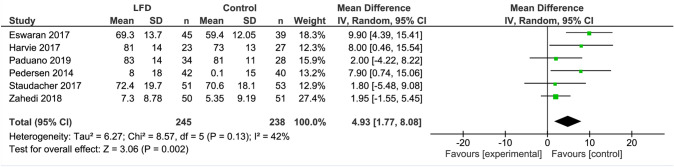
Forest plot showing mean IBS-QoL values

The LFD was associated with higher IBS-QoL scores when compared with a control diet (MD 4.93; 95% CI 1.77, 8.08; *I*^2^ = 42%) (Fig. 4).

#### Subgroup analyses

Subgroup analyses for age, outcome measure, and adherence revealed no statistically significant differences between subgroups (Table 3, Supplementary Figures 1–6). In all studied subgroups, the change in IBS symptom severity scores remained statistically significant (Table 3, Supplementary Figures 1–6).

**Table 3:** Results of subgroup analyses for different covariates

Covariate	Number of studies	Standardized mean difference	LL	UL	*P* value within group	*P* value between subgroups	*I*^2^ (%)
Adherence	–	^–^	–	–	–	0.77	54
Reported adherence^a^	6	− 0.63	− 1.01	− 0.24	0.001	–	66
Adherence not reported	6	− 0.70	− 0.96	− 0.43	0.001	–	42
Age	–	–	–	–	–	0.40	54
Below median^b^	6	− 0.76	− 1.09	− 0.43	0.001	–	59
Above median^b^	6	− 0.56	− 0.87	− 0.25	0.001	–	52
Duration	–	–	–	–	–	0.59	50
Median^c^	5	− 0.53	− 0.88	− 0.18	0.003	–	61
Above median^c^	5	− 0.65	− 0.93	− 0.37	0.001	–	39
IBS subtype	–	–	–	–	–	–	–
Majority IBS-D	6	− 0.62	− 0.84	− 0.39	0.001	–	30
Outcome measure	–	–	–	–	–	0.28	13.9
IBS-SSS	6	− 0.56	− 0.85	− 0.27	0.001	–	61
Non-IBS-SSS	6	− 0.81	− 1.16	− 0.46	0.001	–	44
Risk of bias	–	–	–	–	–	–	–
Low risk of bias	9	− 0.66	− 0.92	− 0.40	0.001	–	55

*IBS* irritable bowel syndrome; *IBS-D* irritable bowel syndrome with diarrhea; *IBS-SSS* IBS symptom severity score; *LL* lower level of 95% confidence interval; *UL* upper level of 95% confidence interval

^a^Adherence was good in all studies that reported adherence

^b^Median age was 40.9 years

^c^Median duration was 4 weeks

#### Sensitivity analysis, assessment of potential biases, and heterogeneity

Sensitivity analyses, conducted by omitting every study from the meta-analysis, were carried out and did not significantly affect the results (Supplementary Tables 1 and 2). Overall, all included studies had some risk of bias, most notably assessed unclear in allocation concealment and blinding of participants, personnel and of outcome assessment (Supplementary Table 3). Three studies were judged to have a high risk of bias in at least two out of seven areas [41, 52, 53], which all at least include blinding of participants, personnel, and of outcome assessment. Excluding these studies in a subgroup analysis did not affect the SMD (Table 3).

**Fig. 5:**
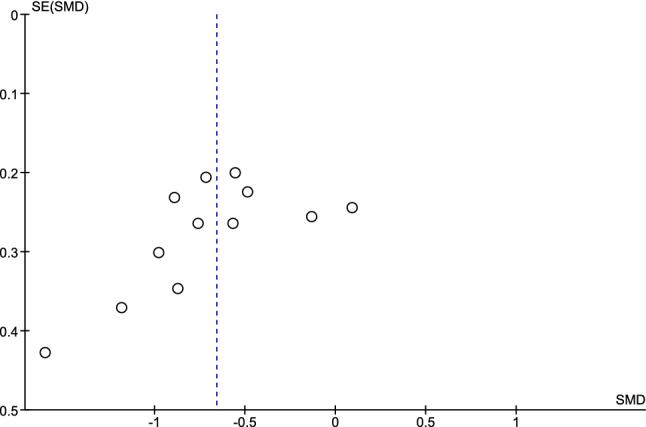
Funnel plot used to assess risk of publication bias for IBS severity outcome measures

Visual inspection of the funnel plot suggested some publication bias (Fig. 5), which was confirmed by Egger’s regression test (*P* = 0.087). The pooled IBS severity measure differences showed moderate heterogeneity (*I*^2^ = 54%) between studies.

### Qualitative analysis

Overviews of the systematic searches for studies investigating the effects of the LFD on gut microbiome and nutritional adequacy are presented in Supplementary Figures 7 and 8. For both outcomes, seven studies met the inclusion criteria and were included in the qualitative analysis.

#### Gut microbiome effects

The methodology employed for fecal microbial analyses varied across studies and included fluorescence in situ hybridization (FISH), quantitative real-time PCR and 16 s rRNA sequencing or combinations thereof.

Five of the included studies reported measures of microbial diversity, six studies reported absolute or relative abundances of total bacteria and/or specific taxa and two studies determined a “dysbiosis index”. In all five studies that measured it, no influence of the LFD measures on microbial diversity was found. However, in most studies, abundances of bifidobacteria and/or their overarching phylum, actinobacteria were reduced following LFD interventions (Table 4).

**Table 4:** Overview of studies assessing the effect of the LFD on gut microbiome

First author, year (country)	Study design	Number of IBS subjects	Intervention	Study duration	Methodology	Results
Halmos, 2015 [31] (Australia)	Crossover	27	LFD vs. baseline habitual diet vs. Australian diet	6 weeks	qPCR	↓ Total bacterial abundance ↓ *A. muciniphila, Bifidobacteria* absolute abundance ↓ *A. muciniphila,* relative abundance ↓ *Clostridium cluster IV* and *XIVa* absolute and relative abundance
Harvie, 2017 [52] (New Zealand)	Parallel	45	LFD vs. habitual diet	12 weeks	16S rRNA sequencing	↔ α- and β-diversity ↔ In any of 244 observed OTUs
Hustoft, 2017 [71] (Norway)	Crossover	27	LFD (maltodextrin supplement) vs. HFD (FOS)	20 days	GA-map™ Dysbiosis Test	vs. baseline: ↓ Actinobacteria abundance ↓ Bifidobacterium abundance ↓ Clostridium, *F. prausnitzii*, Megasphaera, Pediococcus abundance ↑ Dorea abundance
Bennet, 2018 [72] (Sweden)	Parallel	67	LFD vs. traditional IBS diet	4 weeks	GA-map™ Dysbiosis Test	↑ Dysbiosis Index ↓ Actinobacteria abundance ↓ Bifidobacteria abundance
McIntosh, 2017 [64] (Canada)	Parallel	37	LFD vs. HFD diet	3 weeks	16S rRNA sequencing	↔ α- and β-diversity ↑ Acintobacteria richness and diversity ↑ Firmicutes-, clostridiales richness (IBS-D and IBS-M only) ↓ Bifidobacterial relative abundance
Staudacher, 2012 [66] (UK)	Parallel	41	LFD vs. habitual diet	4 weeks	FISH	↔ Concentrations and proportions of total bacteria, *Bacteroides–Prevotella*, *E. rectale–C. coccoides, F. prausnitzii,* and Lactobacillus–Enterococcus ↓ Concentrations and proportions of bifidobacteria
Staudacher, 2017 [69] (UK)	Parallel	104	LFD vs. sham diet	4 weeks	qPCR and 16S rRNA sequencing	↔ α- and β-diversity ↓ Absolute- and relative abundance of bifidobacteria ↔ Relative abundance of lactobacilli and streptococci
Wilson, 2020 [73] (UK)	Parallel	41	LFD vs. sham diet	4 weeks	FISH and 16S rRNA sequencing	↔ α- and β-diversity ↓ Actinobacteria abundance ↔ Bifidobacteria abundance

All reported changes are for LFD vs. respective control situations

*CTRL* controls; *FISH* fluorescence in situ hybridization; *FODMAP* fermentable, oligo-, di-, mono-saccharides and polyols; *GOS* Galacto-oligosaccharides; *HFD* high-FODMAP diet; *IBS* irritable bowel syndrome; *LFD* low-FODMAP diet; *OTUs* operational taxonomic units; *qPCR* quantitative polymerase chain reaction; ↑ increase; ↓ decrease; ↔ no change

#### Nutritional adequacy

Studies reporting on the effects of the LFD on nutrient intake consisted of two post hoc analyses of previous RCTs, three observational studies and two RCTs that only analyzed changes in macronutrient intakes (Table 5).

**Table 5:** Studies included to assess nutritional adequacy of the LFD

First author, year (country)	Study design	Number of IBS subjects	Intervention	Study duration	Methodology	Results
Eswaran, 2019 [56] (USA)	Parallel	78	LFD vs. traditional IBS diet (NICE)	4 weeks	3-day food diary (at baseline and last week of intervention period). Post hoc analysis of [70]	Reduction in energy-adjusted carbohydrate (− 31.6 g/day), total sugar (− 17.4 g/day), sodium (− 0.5 g/day) (all *P* < 0.01) and riboflavin (− 0.2 mg/day) intake (*P* < 0.05) vs. baseline, compared to no changes in traditional IBS diet; increase in energy-adjusted niacin (0.7 mg/day, *P* < 0.05) and vit B6 (0.3 mg/day, *P* < 0.01) intake vs. baseline, compared to no changes in traditional IBS diet; fewer patients met the DRIs for thiamin and iron in the LFD group, vs. fewer patients meeting the DRIs for calcium and copper in the control group
O’Keeffe, 2018 [54] (UK)	Prospective follow-up study	103	LFD vs. habitual diet	6–18 month follow-up after initial 6-week LFD	Semi-quantitative FFQ (at follow-up)	No statistically significant differences between groups at long-term follow-up for energy and (micro)nutrient intakes, except for higher folate (398 µg/day vs. 318 µg/day, P = 0.02) and vitamin A (2147 µg/day vs. 1429 µg/day, *P* = 0.045) compared to habitual diet
Ostgaard, 2012 [55] (Norway)	Prospective follow-up study	114	LFD advice vs. no advice vs. healthy controls	2-year follow-up after LFD advice	Semi-quantitative FFQ (at follow-up)	No difference in calories or macronutrients between LFD guided patients, unguided patients and healthy controls; no difference in micronutrients between LFD guided and unguided patients; lower intakes of riboflavin (1.9 mg/day vs. 2.1 mg/day) and calcium (1065 mg/day vs. 1184 mg/day) and higher intakes of β-carotene (3.9 mg/day vs. 3.6 mg/day) and vitamin B6 (1.7 mg/day vs. 1.6 mg/day) for LFD guided patients vs. healthy controls
Pourmand, 2018 [51] (Iran)	Cross-sectional	3362 (number of confirmed IBS cases NR)	Quintiles of FODMAP intake	–	106-item semi-quantitative food frequency questionnaire	Individuals with the highest adherence to the low FODMAP diet had lower dietary intakes of all measured foods groups and (micro)nutrients (*P* < 0.001)
Staudacher, 2019 [57] (UK)	Parallel	130	LFD vs. habitual diet; LFD vs. sham exclusion diet	4 weeks	7-day food record (at baseline and last week of intervention period); diet quality according to Healthy Diet Indicator and Healthy Diet Score; Diet Diversity according to Diet Quality Index-Revised Dietary Diversity and Dietary Diversity Score Post hoc analysis of [66, 69]	Lower intake of starch vs. habitual control diet (109 g/day vs. 128 g/day, *P* = 0.03); no difference in micronutrient intakes except for higher intake of vitamin B-12 vs. habitual and sham control diets (6.1 μg/day vs. 3.9 μg/day and 4.7 μg/day respectively, *P* < 0.01) and higher intake of selenium vs. sham control diet (52 μg/day vs. 42 μg/day, *P* = 0.03); no difference in proportion of patients meeting micronutrient DRIs; overall scores for diet quality were lower after low FODMAP advice vs. habitual control diet (*P* < 0.01)
*Only macronutrient data*						
Böhn, 2015 [63] (Sweden)	Parallel	67	LFD vs. traditional IBS diet (NICE and BDA)	4 weeks	4-day food diary (at screening and during last week of intervention period)	Reduced mean intake of carbohydrates (159.1 g/day vs. 193.1 g/day, *P* = 0.007) and dietary fiber (15.1 g vs. 20.2 g, *P* = 0.003) vs. traditional IBS diet
Zahedi, 2018 [42] (Iran)	Parallel	101	LFD vs. traditional IBS diet (BDA)	6 weeks	3-day food diary (at baseline and last week of intervention period)	Reduced mean intake of carbohydrates (266.1 g/day vs. 360.9 g/day, *P* < 0.001) and increased mean intake of fat (65.2 g/day vs. 51.4 g/day, *P* = 0.04) vs. traditional IBS diet

*BDA* British Dietetic Association; *DRI* dietary reference intakes; *FFQ* food frequency questionnaire; *FODMAP* fermentable oligo-, di-, monosaccharides and polyols; *GI* gastrointestinal; *IBS* irritable bowel; *LFD* low-FODMAP diet; *NICE* National Institute for Health and Care Excellence; *QoL* quality of life

In most studies, no differences in the majority of analyzed micronutrient intakes were found. Exceptions were small increases in intakes of vitamin A [54], β-carotene [55], B-vitamins [54–57], and selenium [57] after the LFD as compared to control or habitual diets. Conversely, small decreases in riboflavin [55, 56] and calcium [55] intake were also found.

One RCT found that an LFD intervention resulted in a lower proportion of patients meeting the DRIs for thiamin and iron as compared to control [56], whereas a post hoc analysis of two RCTs found no difference in the proportion of subjects meeting micronutrient DRIs when comparing LFD to control diets [57].

One cross-sectional study reported lower intakes of energy, and all measured food groups, macro- and micronutrients across all quintiles of increasing adherence to an LFD [51].

## Discussion

This updated meta-analysis of 12 controlled human intervention studies found that the LFD reduced IBS severity by a moderate to large extent as compared to a control diet (SMD − 0.66, 95% CI − 0.88, − 0.44, *I*^2^ = 54%). Furthermore, the LFD also resulted in higher IBS-QoL scores when compared with a control diet (mean difference 4.93; 95% CI 1.77, 8.08; *I*^2^ = 42%). It should be noted that we used standardized mean differences to include studies that did not use the standard IBS-SSS as an outcome measure. As the SMD can only be interpreted in terms of a small, moderate, or large effect, it limits the extent to which conclusions can be derived about clinical relevance of the demonstrated effect. However, when analyzing only studies that used the IBS-SSS as an outcome measure, a mean reduction of 45 points was found (95% CI − 77, − 14) when comparing subjects on the LFD to a control diet. A 50-point reduction in IBS-SSS score is typically considered to be associated with a clinically meaningful improvement [58]. Nevertheless, the LFD was found to have a moderate to high efficacy in reducing GI symptoms in IBS patients. Our findings are in line with the previous meta-analyses [22, 23, 59, 60], and conclusions are more substantiated due to the higher number of controlled intervention studies that could be included in our analyses (12 controlled intervention studies). The two most recent meta-analyses [22, 23] included only one study and four studies, respectively, to assess the effect of the LFD on QoL. Our review includes six controlled intervention studies that assessed QoL and found a statistically significant 5-point improvement when comparing subjects on an LFD to those on a control diet. Whether this reflects a meaningful change in health-related QoL is unclear, as a 10-point change has previously been considered clinically relevant [35].

In subgroup analyses, we found that the demonstrated improvements in IBS symptom severity were consistent between subgroups with different levels of adherence, age, intervention duration, IBS subtype, outcome measure, and risk of bias. Regarding intervention duration, the longest intervention duration was three months, therefore persistence of symptom reduction may need to be researched further. For IBS subtypes, we only had data to perform a subgroup analysis on IBS with predominantly diarrhea (IBS-D), which revealed outcomes similar to the main analysis. Individual studies with a majority of subjects with IBS with predominantly constipation (IBS-C) [40, 61, 62] or IBS with a mixed stool pattern (IBS-M) [63, 64] generally demonstrated similar improvements in IBS symptom severity, although this was not consistent among all studies [41]. More studies are needed to determine whether the efficacy of the LFD is consistent among these different subtypes. It should be noted that all the subgroups in the current meta-analysis were relatively small and as such the outcomes should be interpreted with caution. Future studies with larger sample sizes and clear reporting on adherence assessment, IBS-QoL assessment, IBS subtype, age, sex, and ethnicity are needed to inform in this regard. Furthermore, there are also no studies that investigated a potential dose–response relationship between FODMAP intake and IBS symptom severity in a controlled systematic fashion, indicating a gap in currently available evidence. However, as the threshold for tolerance of FODMAPs and type of FODMAP varies between individuals, carrying out such study would be very complex. This would likely require a large number of patients recruited in a multicenter setting over a prolonged period of time in a collaborated fashion to be feasible.

All studies had some risk of bias, most notably performance bias due to the lack of blinding of participants, personnel, or outcome assessment. Blinding remains a methodological factor in dietary intervention studies that is very difficult to address, especially in LFD trials where IBS subjects may already be familiar with the LFD due to its increasing popularity. However, a subgroup analysis including only studies with the lowest risk of bias (*n* = 9) did not result in a different SMD as compared to the overall analysis. Furthermore, we found indications of publication bias and visual inspection of the funnel plot suggested an absence of studies reporting a low or no effect on IBS symptom severity.

Owing to the LFDs restrictive nature, concerns have been raised over the long-term nutritional adequacy of the LFD [28, 29, 65, 66], as well as its effects on the gut microbiome [28, 31, 67]. As such, we also examined these aspects as part of the qualitative synthesis of this review (Tables 4 and 5). However, it is difficult to draw definitive conclusions regarding these two outcomes. In both cases, there were only a limited number of studies. Along with heterogeneity in analytical measures and outcome reporting, this precluded meta-analyses or direct comparisons of the available data.

In general, different studies demonstrated that substantial nutritional inadequacies do not occur, both during short-term interventions and at long-term follow-up after initial LFD advice [54–57], and may in some cases even lead to small increases in micronutrient intake. Conversely, a cross-sectional study of a large Iranian cohort did find lower intakes of energy, and all measured food groups, macro- and micronutrients across quintiles of increasing adherence to an LFD [51]. However, it is not clear whether the analyses were corrected for energy intake or other potential confounders.

It is important to note that in most of the included studies, subjects received personalized diets and/or nutritional advice under specialist dietetic or nutritionist guidance, which would have helped to maintain a balanced diet. This underscores the importance of specialist counseling where food items are also reintroduced on a timely basis for IBS patients when following an LFD [26]. Furthermore, although the outcomes of the two included long-term follow-up studies [54, 55] are promising, more work is required to conclusively determine the nutritional impact of LFD in individuals that follow it without seeking specialist advice.

The gut microbiome composition is hypothesized to undergo detrimental changes on an LFD, mainly due to decreased fiber intake and availability of prebiotic fructans, causing a reduction in the substrate available for colonic fermentation [66, 68]. Generally, the LFD did not seem to affect measures of overall microbial diversity, but absolute or relative abundances of actinobacteria were reduced in many cases. Owing to differences in the methodology employed for fecal microbial analyses, it is difficult to compare outcomes between studies. It should also be noted that, since the natural interpersonal variation in gut microbiome composition can result in potentially larger differences than the effect of a dietary intervention, large sample sizes are required to enable robust investigations in this regard. As such, none of the included studies were sufficiently powered to allow for firm conclusions to be drawn. It must also be noted that very few studies have investigated the sustained effects of the LFD on the gut microbiome effects of an LFD (the longest study duration included here was 12 weeks). More work is therefore needed in this regard.

There are some limitations to the current study. First, there was a large variation between studies in control diets, ranging from subjects maintaining their habitual diet without dietary advice to high-FODMAP diets and sham exclusion diets. The FODMAP content of these control diets was often unclear or not reported. The high variety in control diets is also a possible explanation for the moderate heterogeneity observed between studies included in this meta-analysis. Second, half of the included studies did not assess subject adherence to the diet [41, 42, 52, 53, 66, 69]. Other studies assessed adherence via food diaries [40, 62–64, 70] or breath hydrogen tests [61] and reported good adherence. Since adherence is crucial to symptom relief [65], proper reporting in this regard is important to be able to determine the efficacy of an LFD intervention. Also, from a practical point of view, reporting adherence explores the feasibility of following an LFD for IBS patients*.* Nevertheless, subgroup analyses did not reveal significant differences in effect between studies that reported adherence and studies that did not.

In conclusion, this up-to-date systematic review and meta-analysis found that the low-FODMAP diet reduces gastrointestinal symptoms and improves quality of life in IBS subjects when compared to a control diet. Future research is recommended to obtain definitive answers regarding potential long-term effects of such diets on nutritional adequacy and the gut microbiome. This will require larger RCTs with appropriate controls that report on gut microbiome effects, dietary adherence, IBS-QoL and dose–response effects.

## Supplementary Information

Below is the link to the electronic supplementary material.

Supplementary file1 (PDF 477 kb)

Supplementary file2 (PDF 97 kb)

## Data Availability

Data will remain available for 5 years.

## Abbreviations

CI: Confidence interval
FODMAP: Fermentable oligo-, di- and monosaccharides, and polyols
GI: Gastrointestinal
GRADE: Grading of recommendations, assessment, development and evaluations
IBD: Inflammatory bowel disease
IBS: Irritable bowel syndrome
IBS-C: Irritable bowel syndrome with constipation
IBS-D: Irritable bowel syndrome with diarrhea
IBS-M: Irritable bowel syndrome with mixed stool pattern
IBS-U: Unspecified irritable bowel syndrome
IBS-SSS: Irritable bowel syndrome severity scoring system
MD: Mean difference
PRISMA: Preferred reporting items for systematic reviews and meta-analyses
QoL: Quality of life
RCT: Randomized controlled trial
SD: Standard deviation
SE: Standard error
SMD: Standardized mean difference
NR: Not reported
