# Correction to: Efficacy of a low-FODMAP diet in adult irritable bowel syndrome: a systematic review and meta-analysis

**DOI:** 10.1007/s00394-021-02620-1

**Published:** 2021-06-28

**Authors:** Anne-Sophie van Lanen, Angelika de Bree, Arno Greyling

**Affiliations:** 1 Division of Human Nutrition and Health, Wageningen University and Research, Wageningen, The Netherlands; 2 Unilever, Unilever Foods Innovation Centre, Bronland 14, 6708 WH Wageningen, The Netherlands

## Correction to: European Journal of Nutrition 10.1007/s00394-020-02473-0

The original version of this article unfortunatelȳ contained a mistake. The X-axis labels (‘Favours [control]’ and ‘Favours [experimental]’) were presented in the wrong order in Fig. 4.

The corrected Fig. 4 is given below.

**Fig. 4:**
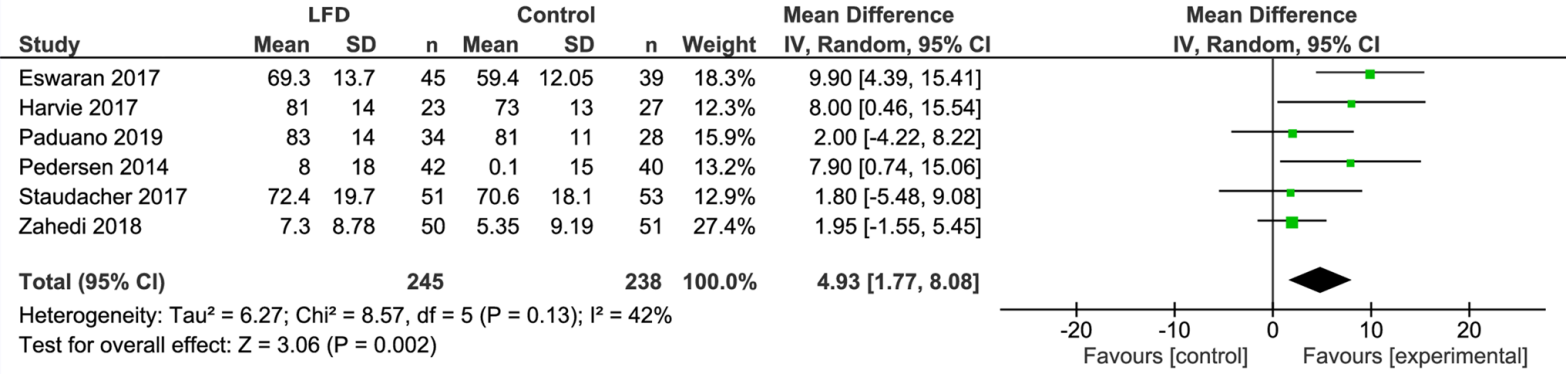
Forest plot showing mean IBS-QoL values

