# Supplementary material for: Efficacy of a low-FODMAP diet in adult irritable bowel syndrome: a systematic review and meta-analysis
Source: Eur J Nutr. 2021 Feb 14;60(6):3505–22. doi: 10.1007/s00394-020-02473-0 (PMC8354978; doi:10.1007/s00394-020-02473-0)
Supplement: Supplementary file 1 — Supplementary file1 (PDF 477 kb) [file 394_2020_2473_MOESM1_ESM.pdf]

Supplementary data

The efficacy of a low-FODMAP diet in adult irritable bowel syndrome: a systematic review and meta-analysis

Anne-Sophie van Lanen[1-2], Angelika de Bree[2], Arno Greyling[2-3]

[1] Wageningen University & Research, Division of Human Nutrition and Health, Wageningen, The Netherlands
[2] Unilever, Unilever Foods Innovation Centre, Wageningen, The Netherlands

Address correspondence to: Anne-Sophie van Lanen, anne-sophie.vanlanen@wur.nl

Supplementary materials

Table S1 Sensitivity analysis for the primary analysis

Study out	Standardized mean difference	P value	LL	UL	I^2
Bohn (2015)	-0.71	0.001	-0.90	-0.52	32%
Eswaran (2016)	-0.68	0.001	-0.92	-0.43	58%
Halmos (2014)	-0.67	0.001	-0.91	-0.43	58%
Harvie (2017)	-0.63	0.001	-0.86	-0.40	56%
McIntosh (2017)	-0.64	0.001	-0.88	-0.41	57%
Ong (2010)	-0.60	0.001	-0.81	-0.40	46%
Paduano (2019)	-0.70	0.001	-0.93	-0.48	50%
Patcharatrakul (2019)	-0.65	0.001	-0.89	-0.41	58%
Pedersen (2014)	-0.63	0.001	-0.87	-0.40	56%
Staudacher (2012)	-0.62	0.001	-0.85	-0.40	54%
Staudacher (2017)	-0.67	0.001	-0.92	-0.43	58%
Zahedi (2018)	-0.66	0.001	-0.90	-0.41	58%

Abbreviations: LL, lower limit of 95% confidence interval; UL, upper limit of 95% confidence interval

Table S2 Sensitivity analysis for the secondary analysis

Study out	Mean difference	P value	LL	UL	I^2
Eswaran (2017)	3.87	0.02	0.71	7.03	21%
Harvie (2017)	5.09	0.02	0.90	9.28	58%
Paduano (2019)	6.39	0.004	2.00	10.77	58%
Pedersen (2014)	5.08	0.02	0.85	9.30	58%
Zahedi (2018)	6.99	0.001	3.39	10.58	18%

Abbreviations: LL, lower limit of 95% confidence interval; UL, upper limit of 95% confidence interval

Table S3 Risk of bias table for included studies

Name of first author (year)	Random sequence generation (selection bias)	Allocation concealment (selection bias)	Blinding of participants and personnel (performance bias)	Blinding of outcome assessment (detection bias)	Incomplete outcome data (attrition bias)	Selective reporting	Other bias
Bohn (2015)	Low risk	Low risk	Unclear risk	Unclear risk	Low risk	Low risk	Low risk
Eswaran (2016)	Low risk	Unclear risk	Unclear risk	Unclear risk	Low risk	Low risk	Low risk
Halmos (2014)	Low risk	Unclear risk	Unclear risk	Unclear risk	Low risk	Low risk	Low risk
Harvie (2017)	Low risk	Unclear risk	High risk	High risk	Low risk	Low risk	Low risk
McIntosh (2017)	Low risk	Low risk	Unclear risk	Unclear risk	Low risk	Low risk	Low risk
Ong (2010)	Low risk	Unclear risk	Unclear risk	Unclear risk	Low risk	Low risk	Low risk
Paduano (2019)	High risk	High risk	High risk	High risk	High risk	High risk	Low risk
Patcharatrakul (2019)	Unclear risk	Unclear risk	Unclear risk	Unclear risk	Low risk	Low risk	Low risk
Pedersen (2014)	Low risk	Unclear risk	High risk	High risk	Low risk	Low risk	Low risk
Staudacher (2012)	Low risk	Low risk	Unclear risk	Unclear risk	Low risk	Low risk	Low risk
Staudacher (2017)	Low risk	Low risk	Unclear risk	Unclear risk	Low risk	Low risk	Low risk
Zahedi (2018)	Low risk	Unclear risk	Unclear risk	Unclear risk	Low risk	Low risk	Low risk

Supplementary data

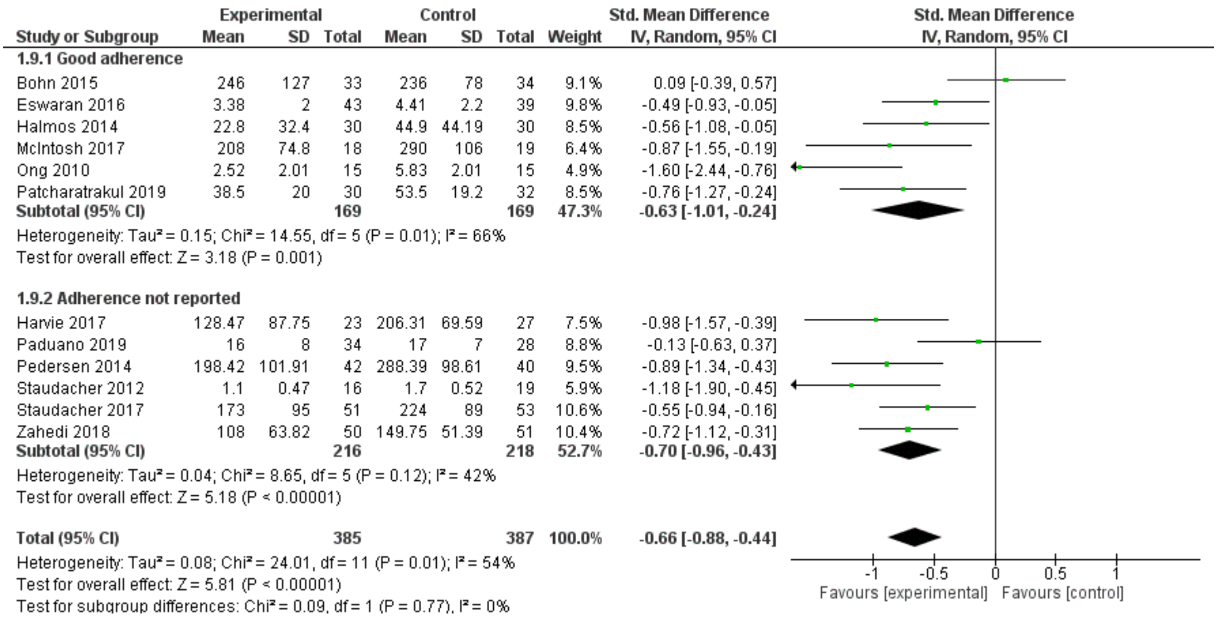

Fig. S1 Forest plot for subgroup analysis on adherence

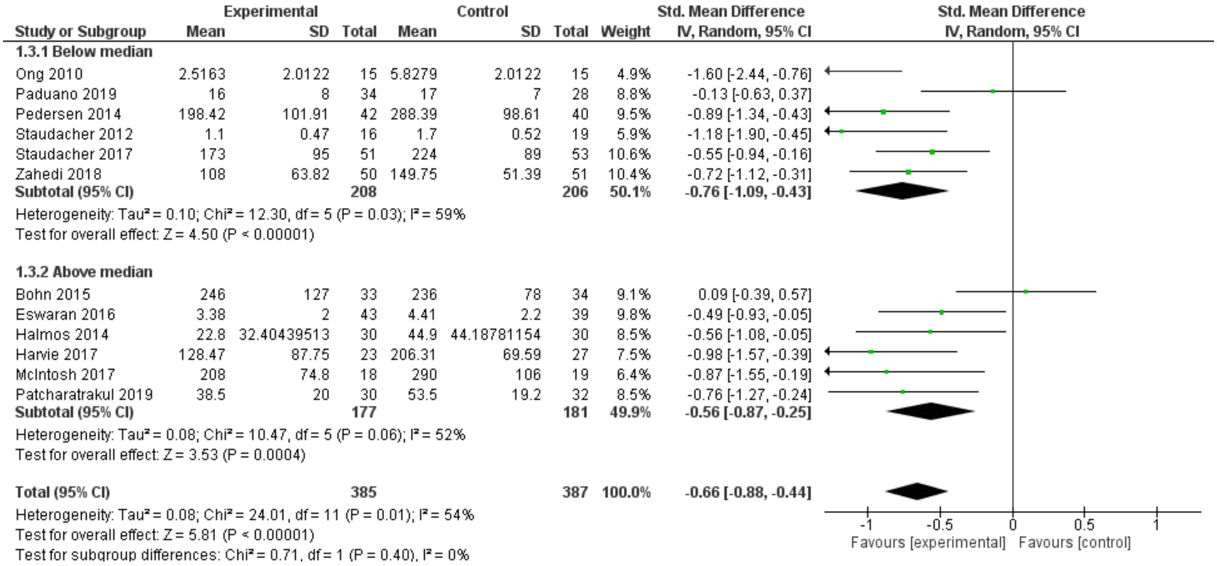

Fig. S2 Forest plot for subgroup analysis on age

Supplementary data

Study or Subgroup	Experimental Mean	Experimental SD	Experimental Total	Control Mean	Control SD	Control Total	Weight	Std. Mean Difference IV, Random, 95% CI
**1.13.1 Median**								
Bohn 2015	246	127	33	236	78	34	10.3%	0.09 [-0.39, 0.57]
Eswaran 2016	3.38	2	43	4.41	2.2	39	11.2%	-0.49 [-0.93, -0.05]
Patcharatrakul 2019	38.5	20	30	53.5	19.2	32	9.5%	-0.76 [-1.27, -0.24]
Staudacher 2012	1.1	0.47	16	1.7	0.52	19	6.2%	-1.18 [-1.90, -0.45]
Staudacher 2017	173	95	51	224	89	53	12.3%	-0.55 [-0.94, -0.16]
**Subtotal (95% CI)**			**173**			**177**	**49.6%**	**-0.53 [-0.88, -0.18]**
Heterogeneity: Tau² = 0.10; Chi² = 10.25, df = 4 (P = 0.04); I² = 61%								
Test for overall effect: Z = 2.94 (P = 0.003)								
**1.13.2 Above median**								
Halmos 2014	22.8	32.40439513	30	44.9	44.18781154	30	9.5%	-0.56 [-1.08, -0.05]
Harvie 2017	128.47	87.75	23	206.31	69.59	27	8.2%	-0.98 [-1.57, -0.39]
Paduano 2019	16	8	34	17	7	28	9.8%	-0.13 [-0.63, 0.37]
Pedersen 2014	198.42	101.91	42	288.39	98.61	40	10.8%	-0.89 [-1.34, -0.43]
Zahedi 2018	108	63.82	50	149.75	51.39	51	12.1%	-0.72 [-1.12, -0.31]
**Subtotal (95% CI)**			**179**			**176**	**50.4%**	**-0.65 [-0.93, -0.37]**
Heterogeneity: Tau² = 0.04; Chi² = 6.58, df = 4 (P = 0.16); I² = 39%								
Test for overall effect: Z = 4.58 (P < 0.00001)								
**Total (95% CI)**			**352**			**353**	**100.0%**	**-0.59 [-0.80, -0.37]**
Heterogeneity: Tau² = 0.06; Chi² = 17.87, df = 9 (P = 0.04); I² = 50%								
Test for overall effect: Z = 5.29 (P < 0.00001)								
Test for subgroup differences: Chi² = 0.28, df = 1 (P = 0.59), I² = 0%								

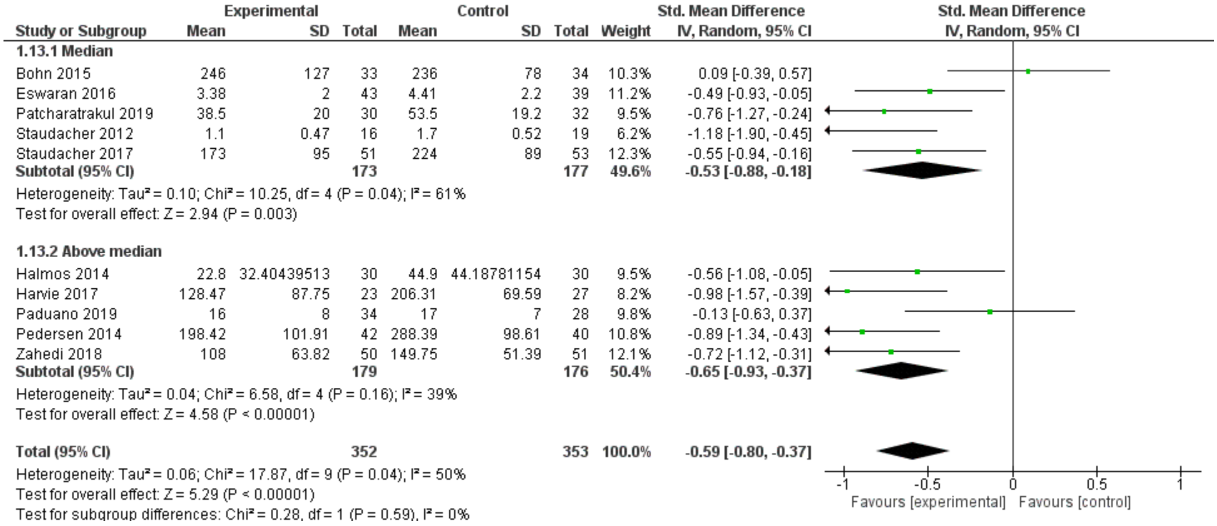

Fig. S3 Forest plot for subgroup analysis on duration

Study or Subgroup	Experimental Mean	Experimental SD	Experimental Total	Control Mean	Control SD	Control Total	Weight	Std. Mean Difference IV, Random, 95% CI
**1.12.1 Majority IBS-D**								
Eswaran 2016	3.38	2	43	4.41	2.2	39	17.5%	-0.49 [-0.93, -0.05]
Harvie 2017	128.47	87.75	23	206.31	69.59	27	11.2%	-0.98 [-1.57, -0.39]
Paduano 2019	16	8	34	17	7	28	14.5%	-0.13 [-0.63, 0.37]
Pedersen 2014	198.42	101.91	42	288.39	98.61	40	16.7%	-0.89 [-1.34, -0.43]
Staudacher 2017	173	95	51	224	89	53	20.4%	-0.55 [-0.94, -0.16]
Zahedi 2018	108	63.82	50	149.75	51.39	51	19.7%	-0.72 [-1.12, -0.31]
**Subtotal (95% CI)**			**243**			**238**	**100.0%**	**-0.62 [-0.84, -0.39]**
Heterogeneity: Tau² = 0.02; Chi² = 7.10, df = 5 (P = 0.21); I² = 30%								
Test for overall effect: Z = 5.45 (P < 0.00001)								
**Total (95% CI)**			**243**			**238**	**100.0%**	**-0.62 [-0.84, -0.39]**
Heterogeneity: Tau² = 0.02; Chi² = 7.10, df = 5 (P = 0.21); I² = 30%								
Test for overall effect: Z = 5.45 (P < 0.00001)								
Test for subgroup differences: Not applicable								

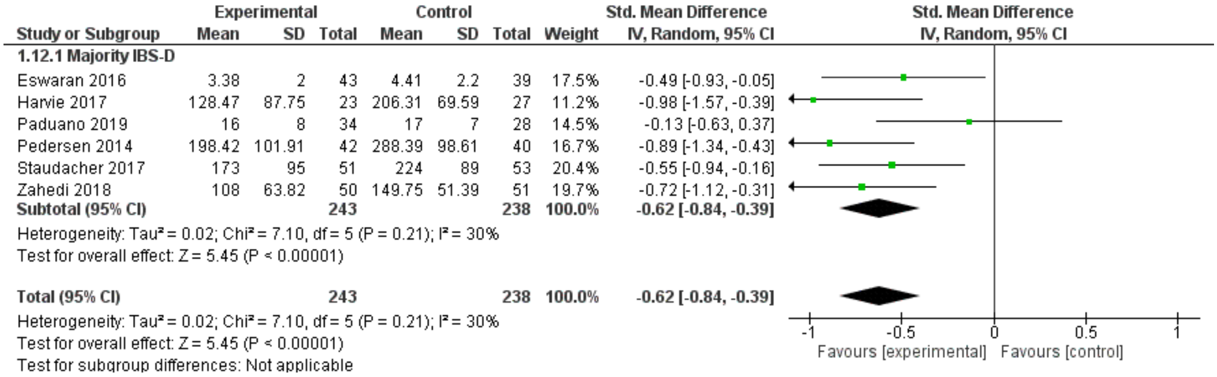

Fig. S4 Forest plot for subgroup analysis on IBS subtype

Study or Subgroup	Experimental Mean	Experimental SD	Experimental Total	Control Mean	Control SD	Control Total	Weight	Std. Mean Difference IV, Random, 95% CI
**1.6.1 non-IBS-SSS**								
Eswaran 2016	3.38	2	43	4.41	2.2	39	9.8%	-0.49 [-0.93, -0.05]
Halmos 2014	22.8	32.4	30	44.9	44.19	30	8.5%	-0.56 [-1.08, -0.05]
Ong 2010	2.52	2.01	15	5.83	2.01	15	4.9%	-1.60 [-2.44, -0.76]
Patcharatrakul 2019	38.5	20	30	53.5	19.2	32	8.5%	-0.76 [-1.27, -0.24]
Staudacher 2012	1.1	0.47	16	1.7	0.52	19	5.9%	-1.18 [-1.90, -0.45]
**Subtotal (95% CI)**			**134**			**135**	**37.6%**	**-0.81 [-1.16, -0.46]**
Heterogeneity: Tau² = 0.07; Chi² = 7.19, df = 4 (P = 0.13); I² = 44%								
Test for overall effect: Z = 4.58 (P < 0.00001)								
**1.6.2 IBS-SSS only**								
Bohn 2015	246	127	33	236	78	34	9.1%	0.09 [-0.39, 0.57]
Harvie 2017	128.47	87.75	23	206.31	69.59	27	7.5%	-0.98 [-1.57, -0.39]
McIntosh 2017	208	74.8	18	290	106	19	6.4%	-0.87 [-1.55, -0.19]
Paduano 2019	16	8	34	17	7	28	8.8%	-0.13 [-0.63, 0.37]
Pedersen 2014	198.42	101.91	42	288.39	98.61	40	9.5%	-0.89 [-1.34, -0.43]
Staudacher 2017	173	95	51	224	89	53	10.6%	-0.55 [-0.94, -0.16]
Zahedi 2018	108	63.82	50	149.75	51.39	51	10.4%	-0.72 [-1.12, -0.31]
**Subtotal (95% CI)**			**251**			**252**	**62.4%**	**-0.56 [-0.85, -0.27]**
Heterogeneity: Tau² = 0.09; Chi² = 15.27, df = 6 (P = 0.02); I² = 61%								
Test for overall effect: Z = 3.75 (P = 0.0002)								
**Total (95% CI)**			**385**			**387**	**100.0%**	**-0.66 [-0.88, -0.44]**
Heterogeneity: Tau² = 0.08; Chi² = 24.01, df = 11 (P = 0.01); I² = 54%								
Test for overall effect: Z = 5.81 (P < 0.00001)								
Test for subgroup differences: Chi² = 1.16, df = 1 (P = 0.28), I² = 13.9%								

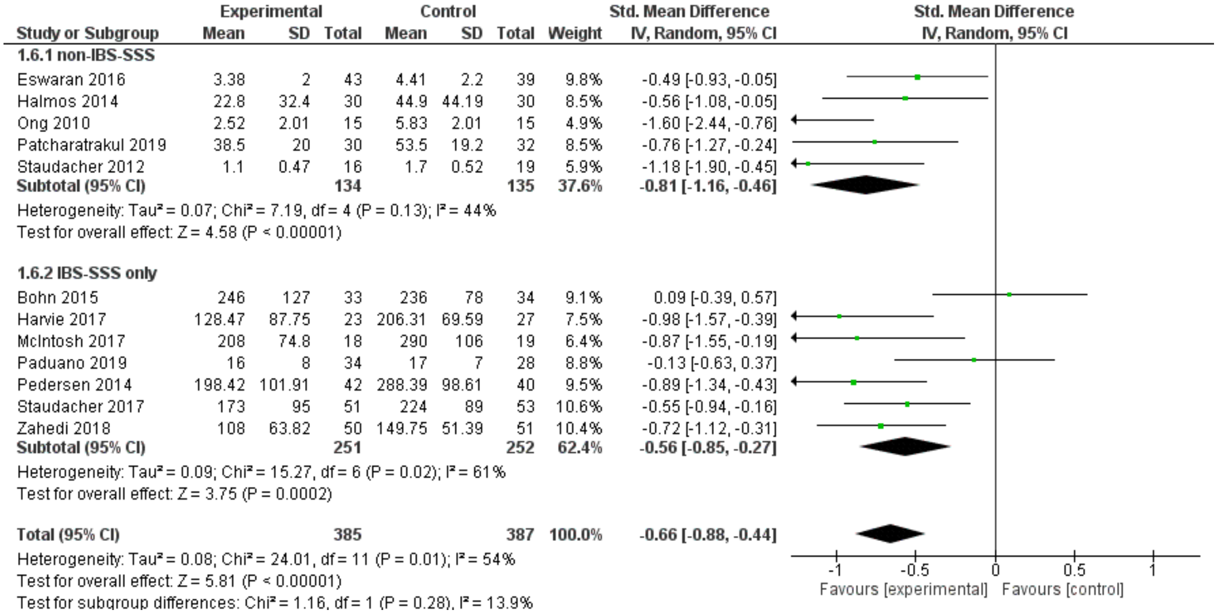

Fig. S5 Forest plot for subgroup analysis on outcome measure

Study or Subgroup	Experimental Mean	Experimental SD	Experimental Total	Control Mean	Control SD	Control Total	Weight	Std. Mean Difference IV, Random, 95% CI
**1.14.1 Low risk of bias**								
Bohn 2015	246	127	33	236	78	34	12.3%	0.09 [-0.39, 0.57]
Eswaran 2016	3.38	2	43	4.41	2.2	39	13.2%	-0.49 [-0.93, -0.05]
Halmos 2014	22.8	32.4	30	44.9	44.19	30	11.5%	-0.56 [-1.08, -0.05]
McIntosh 2017	208	74.8	18	290	106	19	8.6%	-0.87 [-1.55, -0.19]
Ong 2010	2.52	2.01	15	5.83	2.01	15	6.6%	-1.60 [-2.44, -0.76]
Patcharatrakul 2019	38.5	20	30	53.5	19.2	32	11.5%	-0.76 [-1.27, -0.24]
Staudacher 2012	1.1	0.47	16	1.7	0.52	19	7.9%	-1.18 [-1.90, -0.45]
Staudacher 2017	173	95	51	224	89	53	14.3%	-0.55 [-0.94, -0.16]
Zahedi 2018	108	63.82	50	149.75	51.39	51	14.0%	-0.72 [-1.12, -0.31]
**Subtotal (95% CI)**			**286**			**292**	**100.0%**	**-0.66 [-0.92, -0.40]**
Heterogeneity: Tau² = 0.08; Chi² = 17.59, df = 8 (P = 0.02); I² = 55%								
Test for overall effect: Z = 4.98 (P < 0.00001)								
**Total (95% CI)**			**286**			**292**	**100.0%**	**-0.66 [-0.92, -0.40]**
Heterogeneity: Tau² = 0.08; Chi² = 17.59, df = 8 (P = 0.02); I² = 55%								
Test for overall effect: Z = 4.98 (P < 0.00001)								
Test for subgroup differences: Not applicable								

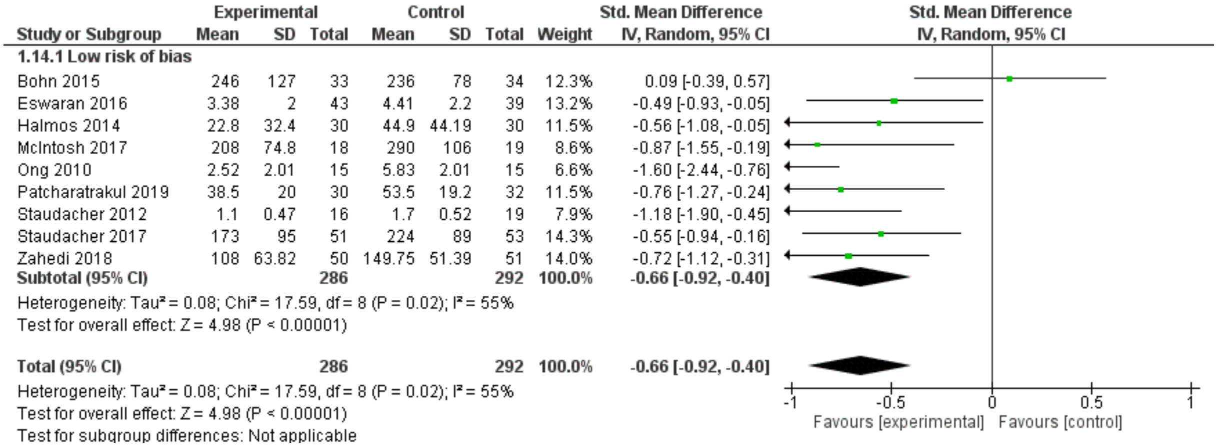

Fig. S6 Forest plot for subgroup analysis on risk of bias

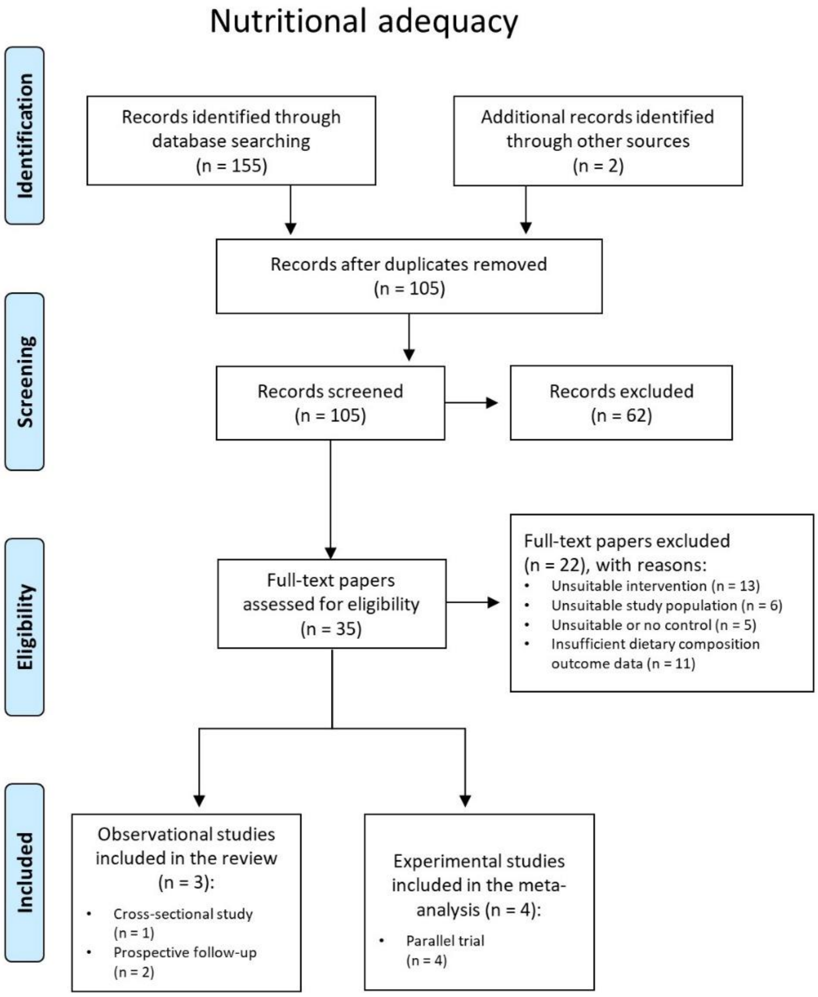

Fig. S7 Preferred Reporting Items for Systematic Reviews and Meta-Analyses (PRISMA) flow diagram of the study selection procedure for the analysis on nutritional adequacy effects

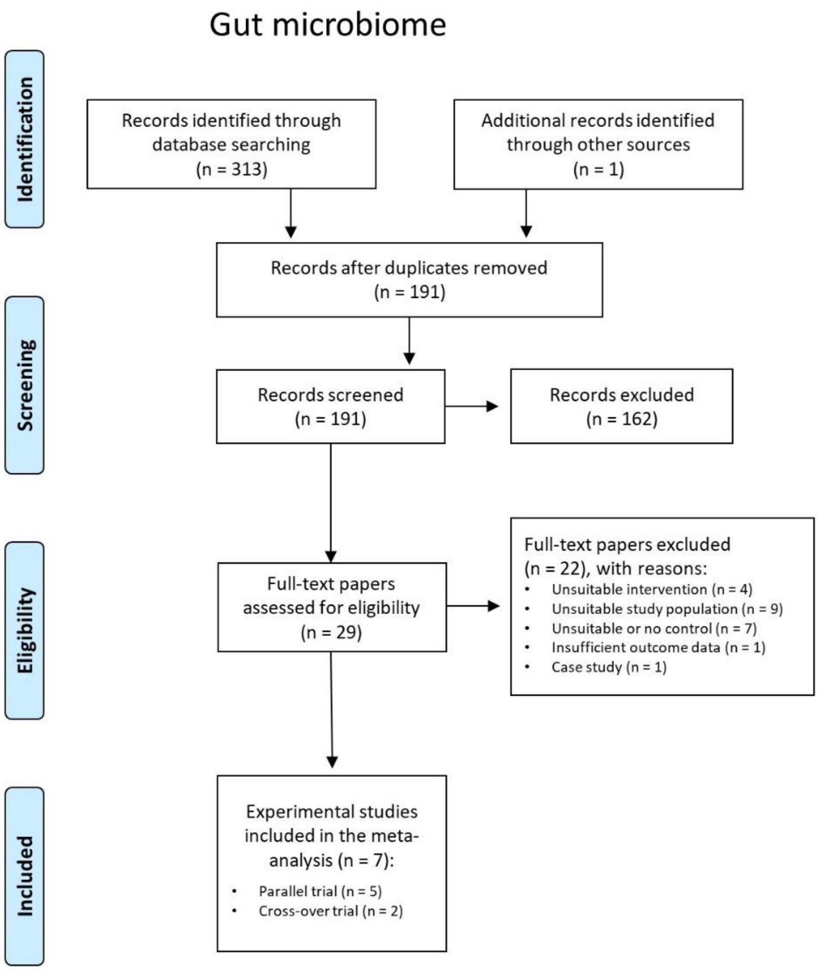

Fig. S8 Preferred Reporting Items for Systematic Reviews and Meta-Analyses (PRISMA) flow diagram of the study selection procedure for the analysis on gut microbiome effects

PubMed search syntaxes

(FODMAP OR FODMAPS OR saccharides OR oligosaccharide OR disaccharide or monosaccharide OR polyol OR polyols OR galacto-oligosaccharides OR fructans OR fructose OR galactans OR lactose OR sorbitol OR mannitol OR xylitol OR maltitol OR sweetener OR sweeteners OR sweetening agent) AND (IBS OR irritable bowel syndrome OR irritable colon)

(FODMAP OR FODMAPS OR "Fermentable Oligo-, Di-, Mono-saccharides And Polyols" OR "low FODMAP diet") AND (microbiota OR microbiome OR "gut microbiota" OR "gut microbiome" OR "gut flora")

(FODMAP OR FODMAPS OR "Fermentable Oligo-, Di-, Mono-saccharides And Polyols" OR "low FODMAP diet") AND (nutrient* OR "nutritional profile" OR "nutrient content" OR "nutrient composition" OR "nutritional content" OR "nutritional composition" OR "nutritional adequacy")
